# Supplementary material for: Are carbon emissions trading and green financial instruments synergistic? -Comprehensive quantitative research based on content analysis
Source: PLoS One. 2024 Mar 7;19(3):e0298601. doi: 10.1371/journal.pone.0298601 (PMC10919601; doi:10.1371/journal.pone.0298601)
Supplement: S1 Data — (ZIP) [file pone.0298601.s001.zip › Data/地级市Z_F工作报告.zip!/╣≤╓▌/╣≤╤⌠╩╨/└┤╫╘╣1⁄2╓┌║┼í╛┬φ┐╦╩2╛▌═°í┐_2017.docx]

各位代表：

　　现在，我代表市人民政府向大会作工作报告，请予审议，并请市政协委员和其他列席人员提出意见。

　　一、过去五年工作回顾

　　2012年以来，在省委、省政府和市委的坚强领导下，在市人大、市政协的监督支持下，我们认真学习贯彻习近平总书记系列重要讲话特别是视察贵州重要讲话精神，始终牢记“守底线、走新路、奔小康”的殷切嘱托，更好地担当全省发展“火车头”“发动机”的历史使命，大力实施主基调主战略，全面推进大扶贫、大数据两大战略行动，以大数据为引领加快打造创新型中心城市，顺利完成市十三届人大一次会议确定的目标任务，各项事业在科学发展中再攀新高。

　　过去五年，我们按下快进键，保持了快速增长势头。经济增速连续4年位居全国省会城市第一，总量超过2个省会城市。2016年，全市生产总值达到3157.7亿元，年均增长14%。规模以上工业增加值达到780.82亿元，年均增长13%；固定资产投资（500万元口径）达到3380.73亿元，年均增长21.5%；社会消费品零售总额达到1195.34亿元，年均增长13.8%；一般公共预算收入达到366.32亿元、支出达到525.61亿元，年均分别增长16.7%和13.6%；金融机构存款余额达到9928.3亿元、贷款余额达到9153.2亿元，分别是2011年末的2.66倍和2.35倍。城镇、农村常住居民人均可支配收入分别达到29502元和12967元。

　　过去五年，我们发展大数据，增强了创新驱动能力。获批建设国家级大数据产业发展集聚区、大数据产业技术创新试验区，成为国家大数据（贵州）综合试验区核心区，首个大数据国家工程实验室落户贵阳。全域公共区域免费WiFi城市一期建成投用，政府数据共享交换平台、开放平台上线运行，大数据安全体系顶层设计初步完成，数据铁笼、大数据综合治税、国有企业大数据监管初见成效，社会和云、智慧城管、智慧交通等一批云应用示范工程投入使用。全市数据中心服务器3.5万台，呼叫中心坐席15万席，大数据及其关联企业达到4000户，产业规模总量超过1300亿元，电子商务交易额达到700亿元，年均增长66.4%。新增国家级、省级工程技术研究中心38家、院士工作站8个，聚集国际国内创新创业人才团队200支，全社会研发投入累计超过150亿元，科技进步对经济增长贡献率提高到58%。鼓励创新、宽容失败的社会氛围日益浓厚，形成全国瞩目的“贵漂”现象。

　　过去五年，我们坚持快转高，推动了产业转型升级。供给侧结构性改革成效明显，“5个100工程”成为经济发展的重要平台，以高新技术产业和现代制造业为龙头的现代工业、以金融业为龙头的现代服务业、以都市农业为龙头的现代农业加快发展，三次产业结构调整为4.3:38.6:57.1。规模以上工业企业全面启动“千企改造、一企一策”，吉利整车制造等一批标志性项目落地建设，装备制造、现代制药等特色工业增加值占规模以上工业比重达74%。获批全国移动金融试点城市、科技金融试点城市，贵州金融城建成投用，全国首家大数据交易所、众筹金融交易所开业运营，贵阳银行A股成功上市，会展业综合排名进入全国省会城市前列，入选国家全域旅游示范区，青岩古镇创建国家5A级旅游景区通过验收，全市年旅游接待突破1亿人次、总收入达1389.51亿元，现代服务业增加值占服务业比重超过52%。创建省级现代高效农业示范园区33个，成功打造农业嘉年华品牌，现代农业增加值占一产比重超过33%。

　　过去五年，我们坚决守底线，巩固了生态文明优势。建设全国生态文明示范城市取得阶段性成效，创建国家环境保护模范城市通过验收，保持“全国文明城市”和“国家卫生城市”称号。“千园之城”建设加快推进，新增各类公园410个，全市公园总数达到705个，成功打造阿哈湖等国家湿地公园。“蓝天”“碧水”“绿地”“清洁”“田园”五项保护计划深入实施，造林绿化127.76万亩，恢复中心城区山体植被6000亩，森林覆盖率提高到46.5%，南明河水环境持续改善，集中式饮用水源水质达标率稳定在100%，环境空气质量优良率提高到95.6%。率先出台建设生态文明城市条例，首创成立生态文明建设委员会，实行生态环境损害党政领导干部问责等制度，以最严的措施、最高的标准，守护了爽爽贵阳的绿水青山，贵阳正以坚实的步伐走向生态文明新时代。

　　过去五年，我们致力增活力，加快了改革开放步伐。“营改增”全面推开，市属平台公司向实体化转型发展，“六统一”土地出让和供给制度全面施行，不动产统一登记大力推进，农村产权制度改革走在全省前列，建立“借、用、还”一体化投融资运行机制。京筑创新驱动区域合作成果丰硕，中关村贵阳科技园成为统揽开放创新的总平台，贵阳综保区应时而生，双龙航空港经济区高起点规划建设，高新区、经开区双双获批长江经济带国家级转型升级示范开发区，构建了“四轮驱动”开放格局。生态文明贵阳会议升格为生态文明贵阳国际论坛，成功打造数博会国际品牌。与美国、瑞士、德国、韩国、印度、台湾、香港等30多个国家和地区广泛开展友好交流与务实合作，累计引进省外到位资金5463.44亿元、实际直接利用外资39.13亿美元，外贸进出口总额310.91亿美元。

　　过去五年，我们推动大建设，改善了城乡环境面貌。城乡规划体系形成“规划树”层级结构，“双核多组团”城市空间布局初步形成。完成棚户区改造1647万平方米，花溪大道汽车市场、市西路批发业态等实现整体转移，贵阳城乡规划展览馆、省博物馆新馆等功能性设施建成投用，“三环十六射”骨干路网全面建成，中环路、首条BRT快速公交系统投入使用，高速公路通车里程突破500公里，提等改造农村公路1441公里，轨道交通1号线、2号线一期建设加快推进，贵阳北站建成运营，市域城际快铁贵开线建成投运，贵阳至广州、长沙、昆明等城市高铁相继开通，龙洞堡国际机场跻身千万级大型繁忙机场行列。一体化大环卫改革扎实推进，整治提升城市干道和背街小巷183条。“四在农家·美丽乡村”六个小康行动计划取得明显成效，农村生产生活环境持续改善，建成美丽乡村提高型示范带15条、示范点185个。

　　过去五年，我们突出高一格，实现了率先全面小康。全市38.35万贫困人口、432个贫困村实现“减贫摘帽”，在全省率先消除绝对贫困，率先以县为单位通过全面小康考核验收，率先实现农村低保标准与扶贫标准“两线合一”，农村低收入困难群体人均可支配收入整体越过4300元。民生实事持续加力、“民生十困”不断破解，累计新增学位13.88万个，新建、改扩建中小学校和幼儿园278所，与北京、上海、山东等地优质学校合作办学116所，建成投用贵州（清镇）职教城。成功创建国家公共文化服务体系示范区，圆满举办国际田联世界越野锦标赛、贵阳国际马拉松赛等重大赛事。新增城镇就业103万人，城乡低保、基本医疗、基本养老等保障标准不断提高，养老服务体系初步建立，新建中心敬老院11所。市医管集团组建成立，贵阳互联网医院投入运营，标准化建设社区卫生服务中心24个、乡镇卫生院67所、村卫生室427个。“两孩政策”全面实施。改造老旧小区院落1500余个、农村危房27597户，开工建设公租房10.16万套、分配8.1万套，建成扶贫生态移民安置房3901套。开工建设中小型水库20座，新建和改造农村饮水安全工程861处，解决农村58.2万人饮水安全。建成高标准蔬菜保供基地2万亩、农产品交易市场14个、惠民生鲜超市23个。被列为全国从整体上加强和创新社会管理三个大城市试点之一，“两严一降”、禁毒人民战争和信访维稳“一号工程”扎实推进，刑事发案数、万人发案数分别下降45.43%和49.11%，信访积案化解率达96.7%，安全生产事故起数、死亡人数持续“双降”，群众安全感上升到98.12%。

　　过去五年，我们持续转作风，加强了政府自身建设。推进政府系统全面从严治党，加强和规范党内政治生活，加强党内监督，切实增强政治意识、大局意识、核心意识、看齐意识。扎实开展党的群众路线教育实践活动、“三严三实”专题教育和“两学一做”学习教育，认真贯彻中央八项规定、省委十项规定，严格执行市委二十九条实施细则，持之以恒转变作风，强化行政监察和审计监督，全面推进民生监督，严查政府机关、事业单位不正之风和违规违纪违法行为，党纪政纪处分1786人。落实权力清单、责任清单制度，公布清单事项4074项，委托或下放行政审批事项67项，全面推行政务公开和公共资源统一集中交易。提请市人大常委会审议地方性法规草案16件，制定政府规章69件，办理人大代表建议993件、政协提案1839件，办复率均达100％。

　　各民主党派、工商联、无党派人士和工会、共青团、妇联、科协、文联、社科联、残联、侨联、红十字会、关工委等为贵阳发展作出新贡献。国家安全、国防动员、民族宗教、司法行政、审计、外事、对台、统计、质量强市、食品药品安全、人防、档案、地方志、机关事务管理、爱国卫生、供销、仲裁、气象等工作取得新进步。

　　刚刚过去的2016年，是“十三五”的开局之年，我们以开局就是决战、起步就要冲刺的劲头，主动把握新常态、积极应对新挑战、有效化解新矛盾，经济社会发展保持了稳中有进、稳中向好的态势，全年生产总值增长11.7%，在全省增比进位综合测评中继续保持第一，六个区包揽全省城区方阵前六，“一市三县”均位列全省县域第一方阵前十。我们打好大数据“突围战”，被评为全国首批大数据产业发展水平五星级城市，在全国率先以一个城市范围的真实网络目标为对象进行攻防测试，《贵阳区块链发展和应用》白皮书正式发布，阳光七星、上海智臻等知名企业相继落户，货车帮、东方世纪等本土企业迅速壮大，朗玛入围“2016年中国互联网企业100强”。我们狠抓供给侧结构性改革，“三去一降一补”扎实推进，为企业降低用电、税费、制度性交易等成本53.09亿元，“千企改造、一企一策”完成技改投资62亿元，中铝“退城进园”氧化铝项目成效明显、电解铝项目顺利推进，老干妈在国内油制辣椒行业的龙头地位更加稳固，益佰制药转型升级实现突破。我们大力构建创新生态链，获批国家小微企业创业创新基地城市示范，跻身2016互联网金融十大创新城市，贵阳大数据创新产业（技术）发展中心、思爱普贵阳大数据应用创新中心等平台成功落户，圆满举办全国首届痛客大赛，“痛客梦工厂”上线运行。我们积极创新社会治理，全国禁毒工作现场会在贵阳召开，创新社会治理案例连续三年获评“全国十佳”，拥军爱军贵阳行动成效明显，连续七次荣获“全国双拥模范城”称号。

　　各位代表，五年来，我们倍受关心、倍受关怀。习近平总书记亲临贵阳，作出重要指示，给予我们极大鼓舞和鞭策。李克强总理先后两次到贵阳视察，还有多位党和国家领导同志给予我们悉心指导。省委常委会专题听取贵阳工作情况汇报，明确要求我们打造创新型中心城市。中央和国家部委、省直部门、兄弟市（州）和贵安新区对贵阳工作大力支持。所有这些，进一步坚定了我们砥砺前行的信心和决心！

　　各位代表，过去五年是贵阳经济社会发展最快最好的时期之一，我们不断完善提升符合贵阳实际的发展道路，取得了诸多来之不易的发展成绩。这一切，是党中央、国务院亲切关怀的结果，是省委、省政府和市委坚强领导的结果，是市人大、市政协监督支持的结果，是全市干部群众共同奋斗的结果。在此，我代表市人民政府，向全市各族人民，向各位人大代表和政协委员，向各民主党派、工商联、无党派人士、各人民团体和各界人士，向驻筑人民解放军、武警官兵和人民警察，向中央及省在筑单位，向市法院、市检察院，以及所有关心支持贵阳建设发展的各位老领导、老同志和各界朋友，表示衷心的感谢!

　　总结五年的工作，我们也清醒地看到存在的问题和不足，主要表现在：实体经济支撑作用不够强，新兴业态基础不够牢，经济增长的质量和效益不够高；科技和人才资源特别是高端人才仍较匮乏，创新驱动的内在活力还不充足；城市管理水平有待提升，交通拥堵、背街小巷“脏乱差”等问题仍较突出；优质公共服务资源供给还有缺口，民生保障水平还不够高；有的干部能力不足而不能为、动力不足而不想为、担当不足而不敢为的问题仍然存在，等等。我们将以更加积极的态度、更加有力的措施，认真加以解决。

　　二、今后五年目标任务

　　随着“一带一路”、长江经济带、京津冀协同发展等国家战略的深入实施，随着贵州建设国家大数据综合试验区、生态文明试验区、内陆开放型经济试验区的加快推进，加之贵阳面临生态环境优良、以交通为重点的基础设施逐步完善、经济新常态下供给侧结构性改革持续发力、政治生态清明“四机叠加”的重大利好，未来五年是我们可以大有作为的战略机遇期。我们坚决拥护以习近平同志为核心的党中央的坚强领导，深入学习习近平总书记系列重要讲话精神，深刻领会习近平总书记关于对党绝对忠诚的谆谆教诲、关于“五位一体”总体布局和“四个全面”战略布局、关于“新发展理念”的重大思想、关于管党治党的最新要求，以及所贯穿的强烈历史使命担当，使之成为全市上下的行动纲领、成为贵阳发展的根本遵循、成为各项工作的实践引领、成为伟大事业的坚强保证、成为实现目标的强大力量。

　　今后五年政府工作总体要求：全面贯彻党的十八大和十八届三中、四中、五中、六中全会精神，以马克思列宁主义、毛泽东思想、邓小平理论、“三个代表”重要思想、科学发展观为指导，深入贯彻习近平总书记系列重要讲话特别是视察贵州重要讲话精神，在省委、省政府和市委的坚强领导下，按照市第十次党代会的战略部署，牢固树立“四个意识”，统筹推进“五位一体”总体布局，协调推进“四个全面”战略布局，切实践行新发展理念，坚守发展生态两条底线，坚持实施主基调主战略，深入推进大扶贫、大数据两大战略行动，当好全省发展“火车头”“发动机”，建设更高水平全面小康，加快打造创新型中心城市。

　　今后五年全市经济社会发展的主要目标：围绕以大数据为引领加快打造创新型中心城市，如期建成大数据综合创新试验区、全国生态文明示范城市、更高水平的全面小康社会，力争进入全国创新型城市行列，创新发展更加强劲、城乡面貌更加靓丽、生态环境更加优化、人民生活更加幸福。全市生产总值年均增长11%左右，总量突破5500亿元，固定资产投资年均增长16%，社会消费品零售总额年均增长11%，城镇、农村常住居民人均可支配收入分别达到45000元和21800元，全社会研发投入占GDP比重达2.2%，科技进步贡献率超过65%，森林覆盖率超过50%，空气质量优良率保持在90%以上。

　　围绕上述目标，今后五年将全力抓好六项重点任务。

　　（一）坚持创新驱动，做强大数据战略引擎，全力以赴增强创新型中心城市发展动能。始终把创新摆在全局发展的核心位置，更加主动拥抱大数据时代、抢抓机遇把握大数据时代、积极创新引领大数据时代，大力发展数字经济，形成富有活力的创新生态链，加速新旧动能接续转换，做强做优国家大数据（贵州）综合试验区核心区。抢占大数据发展制高点，突出政府数据共享开放、大数据安全、区块链技术应用三个重要方向，围绕核心业态、关联业态、衍生业态三个业态，持续巩固数据中心、电子商务、呼叫服务、端产品制造等产业基础，不断做强数据清洗、数据挖掘、数据金融、数据安全等产业核心。大力推进大数据、云计算、物联网、人工智能等在各行业各领域融合运用，推动政府数据共享开放、社会事业数据资源整合、政府监管和社会监督有机结合。引进一批国家、行业、区域数据资源，建成一批大数据应用服务平台，培育一批大数据骨干企业，建设一批大数据众创空间，推出一批大数据先进产品，基本构建大数据全产业链、全服务链、全治理链。探索创新数字经济业态、商业模式、发展路径，区块链场景应用迈出坚实步伐。大数据及其关联产业规模总量年均增长25%以上。加快构建以大数据为引领的现代产业体系，扎实推进供给侧结构性改革，加快大数据与三次产业深度融合，以新理念、新模式推动产业发展，做强实体经济，着力打造“三个姊妹篇”，促进产业链和价值链向中高端跃升。工业经济实现倍增，“千企改造、一企一策”全面完成，工业总产值突破6000亿元，成为全国重要的装备制造基地、西部地区重要的高新技术产业基地、全省军民融合发展的核心区和示范区。“医、养、健、管、游、食”深度融合，大健康医药产业总产值突破1200亿元，建成全省大健康产业创新引领示范区。现代服务业“十百千”工程取得显著成效，现代服务业增加值占服务业比重超过55%，建成区域性电子商务中心城市，力争建成西部科技金融创新城市、互联网金融创新城市。打造世界旅游名城取得突破，形成南北呼应的2个国家5A级旅游景区精品集群，旅游业增加值占GDP比重超过15%，建成国家全域旅游示范区。农业供给侧结构性改革取得积极成效，都市现代农业“接二连三”发展，农业产业体系、生产体系、经营体系不断优化，土地产出率、资源利用率、劳动生产率显著提高，现代农业增加值占一产比重超过45%，村集体经济发展壮大，农业农村发展新动能持续增强，建成全省农文旅一体化先行区。发扬“工匠精神”，培育“百年老店”，让更多贵阳品牌像“老干妈”那样风行天下。以大数据释放创新创业能量，大数据国家工程实验室、国家大数据产业孵化器等平台作用得到充分发挥，主动承担国家和行业重大科研项目，取得一批关键技术成果，聚集一批创新型人才，构建大数据技术与应用领域自主知识产权、规则、标准和法律体系，成为具有全国领先地位和较大国际影响力的大数据创新中心、服务中心、金融中心、应用示范中心，形成全民生产数据、使用数据、利用数据进行创新的文化氛围，努力让每一个人都拥有出彩的机会。

　　（二）坚持改革开放，打造内陆开放新高地，全力以赴激发创新型中心城市动力活力。坚定不移深化改革、扩大开放，加快构建“全方位、宽领域、多层次”的立体开放体系，着力培育体制机制新优势，不断拓展开放发展新空间，努力成为国家内陆开放型经济试验区的制高点。持续推进重点改革，深化工商登记制度改革、财税金融体制改革、投融资体制改革、行政审批制度改革、国资国企改革，支持开展监察体制改革。发挥贵阳综保区海关特殊监管区的平台优势，积极复制推广自由贸易试验区成功经验，探索建立外商投资管理体制改革先行先试区，加快开展服务贸易创新发展试点，大力推进投资贸易便利化，打造诚信化、市场化、国际化、法治化的营商环境。持续创优开放平台，充分发挥中关村贵阳科技园统揽作用，做强“四轮驱动”实体性平台，经开区加快构建新型产业体系，打造千亿量级园区，建成以大数据骨干企业为导向的经济转型发展先行区；高新区引领大数据技术创新，加快大数据产业集聚，打造创新型中心城市示范区；贵阳综保区建设全省国际化引领区，打造内陆开放型经济试验区的“桥头堡”、创新型中心城市的“新引擎”、全国生态文明示范城市的“国际化山地保税新城”；双龙航空港经济区以大数据创新为导向，以综合服务业为指向，建设航空港经济综合试验区。生态文明贵阳国际论坛、数博会等活动性平台国际影响力持续增强，带动对外开放水平全面提升。持续拓展开放空间，深度对接“一带一路”、长江经济带、京津冀协同发展等国家战略，深化京筑创新驱动区域合作，加快建立与长三角、珠三角、环渤海、北部湾等区域的铁路、水路货运和公水联运快速通道，主动融入国际国内市场大循环，在参与国际经济技术交流合作、承接国际资本和沿海产业转移、引进知名企业区域性总部和功能性机构等方面取得新突破，累计引进省外到位资金超过万亿元，实际利用外资超过60亿美元。

　　（三）坚持生态优先，突出“一河百山千园”，全力以赴厚植创新型中心城市生态文明优势。牢固树立绿水青山就是金山银山的理念，坚决守住生态底线，形成南明河人文新景观、百山城乡新形态、千园城市新品质，打造天更蓝、山更绿、水更清、城更靓的美丽家园，深入贯彻国家生态文明试验区（贵州）实施方案，建成全国生态文明示范城市。做足做好“一河”文章，率先实现南明河全流域大数据生态治理，完善雨污分流系统，加强支流治理提升，修复流域自然生态，落实长效管护机制，全面改善水环境质量，建设生态文明韵味浓郁的南明河流域生态长廊，带动全域生态文明建设。做足做好“百山”文章，以城市中心区、机场周边、高速公路沿线为重点，绿色化、景观化、系统化治理山体100个以上，打造连接城乡的绿色廊道27条，完善通风走廊，保护环城林带，构建点、线、面、环相结合的城市绿地系统。做足做好“千园”文章，高标准、高质量、高品质新建各类公园300余个，打造市级示范公园27个，全市公园超过1000个。全面加强山、水、林、田、湖治理保护，整村、整乡、整县连片造林绿化，水体“内源”“外源”污染同步防治，推进大气污染综合整治，让市民群众享有更多生态产品。持续创新生态文明体制机制，把“绿色+”理念贯穿生产生活各领域全过程，建立完善资源总量管控和绿色发展管控制度、绿色发展市场制度，试点开展不同功能区差异化考核评价，加大生态环保执法力度，全力捍卫城市生态空间。

　　（四）坚持城乡统筹，加快新型城镇化进程，全力以赴增强创新型中心城市发展后劲。注重整体谋划，补齐薄弱区域、薄弱领域、薄弱环节短板，推进城乡统筹发展，加快建设特色鲜明、和谐宜居的现代化城市。不断优化空间布局，“规划树”层级结构不断完善，城市总体规划与经济社会发展规划深度融合，生态环境、基础设施、人口资源、公共服务、公共空间等专项规划与控制性详细规划有机衔接，构建智慧化多规协同体系，建立城市发展趋势模型，形成城市规划建设管理的“统一坐标系”，避免“摊大饼”，着力“蒸小笼”，进一步优化“双核多组团”城市发展格局。始终坚持建管并举，继续推动不适宜在老城区保留的办公、服务等功能和批发等业态向外疏解，加快城市新区建设和功能配套。全面打通城区断头路，持续实施农村道路提等改造，建成轨道交通线路4条，开通成贵、渝黔等高铁线路，境内高速公路通车里程达到800公里，建成龙洞堡国际机场三期，完成开阳港提档升级，基本形成现代立体交通体系。建成全光网城市，成为区域信息交换枢纽和信息存储中心。加大中小型骨干水库建设力度，基本解决工程性缺水问题。推进海绵城市、地下综合管廊、城市慢行（绿道）系统建设。深入实施“四在农家·美丽乡村”六个小康行动计划，建成一批示范小城镇、特色村庄、美丽乡村。打造智慧城市，促进大数据、物联网、云计算等现代信息技术与城市管理服务有机融合，精细化、长效化、标准化管理水平不断提升，良好的市容环境卫生秩序成为常态。持续壮大县域经济，坚持以“八个一批”为抓手，推动县域差异化、特色化发展，将云岩区、南明区、观山湖区打造成为创新型中心城市核心区，将花溪区建设成为全域文化旅游创新区，将乌当区建设成为全省大健康产业发展引领示范区，将白云区建设成为大数据社会治理先行试验区，将清镇市打造成为生态文明示范城市样板区，将修文县打造成为阳明心学圣地、特色农业基地、现代医养高地，将息烽县、开阳县建设成为更高水平全面小康示范县。推进南部现代制造业实体经济带、北部高新技术产业实体经济带发展，加快“三区两县”融合发展。

　　（五）坚持普惠均衡，决胜大扶贫战略行动，全力以赴增进创新型中心城市民生福祉。深入践行以人民为中心的发展思想，全面实现“六个确保”“六个提升”“六个增强”，让群众享有更多的获得感和幸福感。坚决打赢脱贫攻坚战，按照高一格脱贫、快一步致富的要求，推进科学治贫、精准扶贫、稳定脱贫，农村低收入困难群体人均可支配收入整体越过6500元，生活保障水平不断提高。结对帮扶的9个贫困县全部实现“摘帽”。努力让市民享有更充分的创业就业机会，建设充分就业城市，保持“城镇零就业家庭”和“农村零转移就业家庭”动态为零，累计实现城乡统筹就业110万人以上。努力让市民享有更稳定的收入，实施七大群体激励计划和六大支撑行动，扩大中等收入群体比重，城乡居民收入差距进一步缩小。努力让市民享有更舒适的居住，坚持住房保障与住宅品质两手抓，让更多群众从住有所居向住有优居提升。努力让市民享有更便捷的出行，不断提高城市建成区平均路网密度，城市公交分担率超过40%，“大数据+交通”实现出行精准服务。努力让市民享有更均衡的教育，普惠性幼儿园实现社区全覆盖，义务教育优质均衡发展，普通高中教育特色优质发展，职业教育内涵式发展，高等教育特色发展，构建终身教育学习体系。努力让市民享有更优质的医疗，将健康融入所有政策，建立更高质量的医疗服务体系，构建起“15分钟城市社区健康服务圈”和“30分钟乡村健康服务圈”，开展国家级医养结合试点，建成全国健康城市。努力让市民享有更公平的社会保障，扩大社会保险覆盖面，推进养老服务业综合改革，完善社会救助体系，发展公益慈善事业，形成更加公平可持续的社会保障体系。努力让市民享有更满意的安全生活，建强社会和云，抓好“数据信访”“数据维稳”，推进“两严一降”和禁毒人民战争常态化、制度化、标准化，构建起社会治安立体防控体系、现代城市防灾减灾、突发事件应急体系，创建国家食品安全示范市，切实防范经济风险、社会风险和自然风险，力争成为国家安全发展示范城市，给全市人民一个稳稳的安全感。

　　（六）坚持文化引领，系统构建“三足鼎筑”，全力以赴提升创新型中心城市人文厚度。充满激情地拥抱城市文化的春天，加快推进文化创新发展，不断提升贵阳城市的文化魅力、文化实力、文化张力。大力弘扬社会主义核心价值观，深入开展“明礼知耻·崇德向善”主题实践活动和各类群众性精神文明创建活动，推进道德教育长效化、文化创作精品化、社会宣传立体化，丰富和深化“知行合一、协力争先”贵阳精神的文化内涵，不断提高市民群众对城市的自豪感、认同感、归属感。大力塑造特色文化品牌，加快推进孔学堂传统文化传承教化基地、中国阳明文化园、阳明历史文化街区建设，着力打造世界级中华传统文化交流展示平台、“老贵阳”文化承载中心和“新贵阳”城市客厅，大力推进民族民间文化内容创新、传播方式创新，让阳明文化、生态文化、民族文化、公共文化、时尚文化名片更加响亮。大力完善公共文化服务体系，持续巩固和扩大国家公共文化服务体系示范区创建成果，实施“文化+”系列行动，打造市区“15分钟文化圈”、城乡“十里文化圈”，成为多彩贵州民族特色文化强省的创新创意传播中心、西部领先的国家级公共文化服务体系示范区、国家级文化科技融合发展示范基地。

　　三、2017年工作安排

　　今年将召开党的十九大和省第十二次党代会，也是实施“十三五”规划的重要一年、推进供给侧结构性改革的深化之年，是新一届市政府履职的开局之年，做好全年工作意义十分重大。我们将认真按照市第十次党代会的战略部署，坚持稳中求进工作总基调，以供给侧结构性改革为主线，大力发展数字经济、旅游经济、绿色经济、县域经济，全面做好稳增长、促改革、调结构、惠民生、防风险各项工作，撸起袖子加油干、迈开步子加快赶，更加注重创新、更加注重改革、更加注重质量、更加注重落实，为加快打造创新型中心城市奠定坚实基础。

　　今年经济社会发展的主要预期目标是：生产总值增长11%，规模以上工业增加值增长10%左右，固定资产投资增长18%，社会消费品零售总额增长11.5%，一般公共预算收入增长8%，城镇、农村常住居民人均可支配收入分别增长9%左右和10%左右，居民消费价格涨幅、节能减排指标控制在省下达目标范围内，森林覆盖率提高到47%，环境空气质量稳定在全国省会城市前列。

　　围绕上述目标，我们将重点抓好以下工作。

　　（一）深度聚焦大数据，在增强创新能力上迈出新步伐。深入实施大数据战略行动,全面推进大数据“十大重点工程”，积极创新应用、创新业态、创新模式。抓好数据产业集聚工程，推进大数据产业生态示范基地、大数据安全产业基地等园区建设，启动智能网联汽车、物联贵阳等项目建设，实施大数据“十百千企业”培育计划，力争新增大数据领军企业10家以上，新建服务外包与呼叫中心产业基地、综合园区10个，呼叫中心建设规模达20万席、签约投运15万席，全市大数据及其关联产业规模总量达到1500亿元以上。抓好数据资源汇聚工程，全面建成公共区域免费WiFi，通信光缆达到12万公里，出省带宽突破6000G，建成国家级互联网骨干直联点。实现市、县两级政府数据在“云上贵州·贵阳平台”汇聚。引进第三方数据存储和云计算服务企业2至3家，建成运营世欣蓝讯数据中心，全市数据中心服务器达到4.5万台。引进国家部委及世界500强企业数据资源2至3家，建设国家发展改革委互联网大数据分析中心贵阳分中心。抓好数据共享开放工程，深入推进政府数据“聚通用”，建立政府数据资源目录体系、共享开放制度体系。建成人口、法人等基础信息共享数据库。实施引领性政府数据共享应用项目50个以上，首批向社会开放主题关联数据包1000个以上。探索建立政府数据契约式开放机制，建成政府数据增值服务平台。抓好大数据助推产业升级工程，搭建中关村贵阳科技园产业云平台、贵阳市中小企业云平台，建设人工智能大数据云服务平台，打造数字工厂。开展智慧商店、智慧商圈示范工作，建设益佰肿瘤大数据信息云平台、贵阳康康慢病互联网医院，打造朗玛“医疗机构+药店+网络平台”互联网医疗模式。启动实施区块链技术场景应用项目5个以上，推进正大小镇、VR小镇、智客小镇等项目落地。新增高新技术企业50户。抓好数据金融工程，建成数控金融项目平台，积极申建国家大数据交易平台。抓好数据铁笼工程，实现市级机关、市属企事业单位全覆盖，建成数据铁笼综合监管平台。抓好数据政务工程，建设大数据国家工程实验室。“互联网+政务”基层覆盖率达100%，大数据综合治税系统区（市、县）全覆盖，完善公安块数据指挥中心，推进城乡规划、市场监测监管、经济运行监测分析、信用信息等数据平台建设。抓好数据民生工程，大数据民生工程一期正式上线运行，接入并提供政府和市场民生服务150项以上。建成生态云一期和人口健康信息云、旅游云。抓好数据扶贫工程，全面建成大数据精准帮扶平台，实现跨部门、跨行业帮扶业务全覆盖。抓好数据立法、安全与标准工程，起草贵阳市数据安全条例，申建国家级数据安全试点城市和大数据安全靶场，做好5项地方标准研制和3项国家标准验证示范。

　　（二）深度聚焦优供给，在促进产业升级上迈出新步伐。扎实推进供给侧结构性改革，改造提升传统产业，加快发展新兴产业，持续做强实体经济，不断提高供给体系质量效率。全力落实“三去一降一补”，深入实施“双退出”行动计划，积极稳妥淘汰落后产能、化解过剩产能。加快房地产去库存，提高棚户区改造货币化安置比例，鼓励和支持农民工在城镇购房，规范发展房屋租赁市场。持续推进降成本，力争为企业降低用电成本、税费成本30亿元，通过风险分担等方式继续降低企业融资成本，发挥货车帮、普洛斯“马上到”国际云服务平台作用，用好物流标准化试点城市专项资金，初步建立标准化托盘循环共用体系，进一步降低企业物流成本。全力提升工业供给能力，大力实施“千企引进”工程，引进500强企业5户和科技含量高、成长性好的企业12户。加快推进“千企改造、一企一策”，贵阳吉利年产30万台整车产业化项目首车下线、年产40万台1.5TD发动机项目开工建设、汽车轮毂及铝制零部件项目加快落地，中铝“退城进园”电解铝项目建成投产。推进开磷、贵钢、黔轮胎等企业加快转型，老干妈技改扩能。加快建设海信电子、力源液压等国家级智能制造示范基地，实施黎阳航空发动机系列、航天电器系列智能制造等军民融合项目。促进骨科中药生产基地建成投产，宝石加工基地等项目落地。新增规模以上工业企业100户，全市工业总产值达到3000亿元。全力提升服务业供给能力，继续完善贵州金融城配套，培育普惠金融、征信数据、区块链金融等新兴金融业态，浦发银行贵阳分行、华夏银行贵阳分行等金融机构入驻办公，全市金融业增加值增长15%。与华润集团开展全面战略合作，优化提升西南国际商贸城，启动打造全市首个综合性现代服务业集聚区。全面启动花溪恒大童世界建设。顺应消费需求变化新趋势，大力发展电子商务，实现交易额920亿元、增长30%。加快建设国家全域旅游示范区，争取成为国家大数据旅游试点城市，持续推进天河潭景区改造提升和乡愁贵州、时光贵州、阳明文化园等项目建设，新推出“一程多站式”国际旅游线路2条，实现旅游接待1.37亿人次、总收入1750亿元。推动大健康与大数据、大旅游融合发展，启动贵阳健康医疗城建设，大健康医药产业增加值增长20%以上。全力提升农业供给能力，以提高农业供给质量为主攻方向，拓展农业产业链价值链，做强特色蔬菜、精品水果、生态畜牧业等优势产业，新增“三品一标”种植面积5万亩以上，力争创建有机产品示范县1个，建设高效农业园区50个以上。大力发展农村电商，促进新型农业经营主体、加工流通企业与电商企业全面对接融合。办好第三届、第四届农业嘉年华活动。全力提高产品和服务质量，深入推进质量提升行动，持续开展“市长质量奖”评定工作，大力实施标准领跑者制度，完善产品质量追溯体系和惩罚性赔偿制度，培育品牌企业5家，打造中国驰名商标2个、贵州省著名商标20个，培育贵州省名牌产品25个以上，力争成功创建全国质量强市示范城市。

　　（三）深度聚焦扩投资，在夯实发展基础上迈出新步伐。把握全市经济仍处于投资拉动的阶段性特征，坚持抓投资就是抓发展，精准加力有效投资，提高投资效率和效益，持续增强发展后劲。扩大投资总量，围绕做长“三块长板”、补齐“三块短板”，加强项目谋划，全市项目库投资规模动态保持在2万亿元以上，全年新开工重大工程和重点项目105个、建成28个，县域经济“八个一批”项目投资超过1400亿元、工业投资超过700亿元、基础设施投资超过540亿元、脱贫攻坚投资超过200亿元，力争全市固定资产投资突破4000亿元。激发民间投资，大力推广政府和社会资本合作模式，积极帮助民营企业争取专项建设基金，用好贵阳贵银城市投资发展基金、贵阳市水务产业发展基金等产业引导基金，谋划推出一批工程包，引导社会资本进入轨道交通、市政建设、教育培训、医疗卫生等领域。加强民营企业“大走访、大服务”，开展“待批项目大提速”活动，确保符合条件的项目尽早建设、尽快达效。力争民间投资占比稳定在50%以上。加快项目建设，开展“项目建设大比拼”活动，精心组织集中开工、现场观摩，完善中央预算内资金项目、国家专项建设基金项目、重点招商项目等清单管理制度，强化土地、资金等要素保障，推动项目快建设，促进投资稳增长。

　　（四）深度聚焦生态美，在全域治理保护上迈出新步伐。以构建“一河、百山、千园”自然生态体系为统揽，加强全流域治理、全区域保护，不断优化生态环境。打好生态建设“攻坚战”，新建各类公园237个，建成市级示范公园7个，实施通道绿化、村寨绿化5000亩以上，完成人工造林12万亩以上。实施山体迹地修复、植被提升6000亩。美化、净化、绿化、亮化中心城区屋顶14万平方米，新增城市绿地100万平方米。打好污染治理“突围战”，加快南明河综合治理，建设流域遥感监测、智慧防汛决策、水资源监控等平台，完成花溪河流域治理工程，实施市西河、贯城河、小黄河、麻堤河等支流治理，开工建设贯城河污水处理厂。加快推进小关湖、黔灵湖和金钟河、洋水河等水体治理。开展226个自然村寨生活污水治理。积极服务引子渡调水工程建设，大力加快红枫湖——花溪水库水源保护连通工程进度。推进“两湖一库”周边农村生活污染、废弃煤矿等综合生态治理。深入实施大气污染防治六大重点工程，大力推广使用清洁能源，淘汰黄标车1.1万辆，打造夜市油烟综合治理示范项目13个。加强噪声治理。打好环境监管“持久战”，加强生态环境网格化监管，建立环境保护督察机制，推广第三方治理模式，实行政府、民间“双河长制”，严格环境质量排位考核，持续开展森林保护“六个严禁”和环境保护“六个一律”利剑行动，对环境违法行为“零容忍”。

　　（五）深度聚焦抓改革，在增强发展活力上迈出新步伐。坚持以深化改革促进开放，以扩大开放倒逼改革。扎实推进各项改革，深化“放、管、服”改革，推进“五证合一、一照一码”，实现“双随机、一公开”全覆盖，力争全年市场主体增长12%以上、注册资本总量增长45%以上。深化国有企业改革，加快平台公司实体化转型。创新投融资体制，发挥市级PPP项目管理中心、国有资产运营服务中心作用，组建国有资本控股公司，积极探索规范的市场化融资渠道。全面推进农村“三变”改革，深化供销合作社综合改革，稳步推进机关事业单位养老保险制度改革，扎实推进安全生产领域改革，继续抓好公立医院综合改革。大力提升开放水平，推动高新区、经开区在全国排位前移，贵阳综保区、双龙航空港经济区加快发展，办好系列重大活动，力争数博会成为国家级博览会，持续扩大生态文明贵阳国际论坛成果。加强与欧美、东亚、东南亚地区主要国家的合作，力争落地一批标志性项目。持续深化京筑创新驱动区域合作，在北京、上海等城市建设大数据创新异地孵化器。推进外资、外贸、外经融合发展，确保对外贸易回稳趋好。全年省外到位资金超过1600亿元，实际直接利用外资超过13亿美元。

　　（六）深度聚焦新形态，在提升城市品质上迈出新步伐。大力实施城市规划建设管理“10项行动计划”，提升城市颜值气质品质。突出规划引领，完成城市总体规划修改，完善城市土地利用总体规划，启动编制新一轮教育、体育、公共交通等公共服务设施专项规划。制定中心城区精品街区选址规划、景观控制修建性详细规划方案。出台“一市三县”城乡规划管理指导意见，启动编制镇（乡）村建筑风貌导则。加大建设力度，实施交通基础设施建设“大会战”，抓好新东二环、新北二环一期、贵惠大道、公园路等道路建设，推进5条高速公路建设，实施农村公路提等升级300公里。轨道交通1号线土建工程全面完成、观山湖段试运行，2号线二期全面开工，3号线一期和S2号线一期北段启动建设，渝黔高铁建成通车，加快西南商贸服务业集聚区、龙洞堡机场等综合交通枢纽建设，着力推进海绵城市和地下综合管廊建设。新建公共智慧停车场3个。启动棚户区改造3.3万套，基本建成1.9万套，提升老旧居民小区200个以上。推进小关片区、三马片区、乌当奶牛场片区开发建设。加大传统村落、民居和建筑保护力度，启动历史文化特色街区建设，建成美丽乡村提高型示范点40个。强化精细管理，深入推进城市执法体制改革，一体化大环卫模式“一市三县”全覆盖，推广互联网监控探头视频直播，实现城市环境秩序全方位监管。深入开展城市、农村环境综合整治，完成200条背街小巷和138个村整治工作。以BRT快速公交系统开通为契机，大力发展城市公共交通，规范网约车市场，促进出租车新旧业态融合发展。大力开展治堵保畅专项执法行动，对火车站、贵阳北站、金阳客车站、机场等重点场站，市区和贵阳各进出口重点路段，一环内及花果园、未来方舟等重点区域，加强交通秩序管理、环境卫生整治、非法营运打击等联合执法，打造少堵通畅、干净整洁、平安有序的城市环境，努力让市民群众出行通畅、心情舒畅。

　　（七）深度聚焦补短板，在增进民生福祉上迈出新步伐。政府工作的根本目的，就是让人民群众过上好日子，虽然财政收入增长有所放缓，但该办的民生实事一件也不能少。我们将扎实推进大扶贫战略行动，狠抓产业扶贫、易地搬迁扶贫，继续开展民营企业“千企帮千村”活动，实施20个特别困难村扶贫解困定点包干工作，确保全市农村低收入困难群体人均可支配收入整体越过4800元，100个低收入困难村人均可支配收入增幅高于全市平均水平3个百分点。继续抓好结对帮扶省内贫困县工作。我们将纵深推进“教育立市”战略，巩固扩大京筑教育合作成果，率先在全省启动县域义务教育优质均衡试点，鼓励发展本地优质教育资源，新建、改扩建学校、幼儿园60所，全面完成1个社区1所公办或普惠性幼儿园建设任务，推动贵州（清镇）职教城内涵式发展。我们将大力建设健康城市，创建国家级、省级、市级卫生乡镇15个以上、卫生村寨80个以上，加快县级妇幼保健机构标准化建设，新增甲级乡镇卫生院12所，加强基层卫生人才队伍建设。广泛开展全民健身活动，办好系列品牌体育赛事，建成村级全民健身工程60个。我们将积极促进创业就业，全面启动创建充分就业城市工作，进一步提高职业技能培训质量，城镇新增就业23万人。我们将织密社会保障网，全面实施全民参保计划，扎实推进居民基本医疗保险城乡统筹工作，实现跨省异地就医直接即时结算，完善社会救助标准与物价上涨挂钩联动机制，城乡最低生活保障标准提高10%以上。进一步提升养老服务水平。我们将持续加强文化惠民，全面推进公共文化机构免费开放，依托公共文化设施开展学雷锋志愿服务活动。我们将持之以恒抓好“菜篮子”工程，启动贵阳北部农产品电商物流园建设，新建高标准蔬菜保供基地1万亩、异地联动建设10万亩，建成惠民生鲜超市20个。突出优质、安全、绿色导向，全面提升农产品质量和食品安全水平。大力推进农超对接、农商互联、农校对接，创新流通体制机制，减少中间环节，带动更多贫困群众增收脱贫，为市民群众提供物美价廉的绿色优质农产品。

　　（八）深度聚焦促和谐，在创新社会治理上迈出新步伐。巩固提升城市社区治理成果，探索创新农村基层治理模式，做强居（村）委会，做精网格，推进社会治理社会化、法治化、智能化。严格规范政府举债行为，严厉打击非法集资、金融诈骗，守住不发生系统性区域性风险的底线。深化安全城市建设，深入推进重大事项社会稳定风险评估，持续开展“两严一降”和禁毒人民战争，做好信访维稳工作，完善城市公共安全体系和应急管理体系，提高突发事件快速响应和应急处置能力，抓好地质灾害、城市老旧住房等隐患排查治理，加强重点行业、重点领域安全防范，严防重大群体性事件、重大网络舆情、重大刑事案件、重特大安全事故发生。

　　继续做好国防动员、双拥共建和后备力量建设工作。支持军队、武警部队建设和法院、检察院工作。推进国家安全、民族宗教、司法行政、人口计生、审计、外事、对台、新闻出版、食品药品安全、人防、档案、地方志、爱国卫生、供销、仲裁、气象等工作发展。支持工会、共青团、妇联、科协、文联、社科联、残联、侨联、红十字会、关工委等做好工作。

　　四、扎实抓好政府自身建设

　　深入贯彻落实党的十八届六中全会精神，坚定不移推进全面从严治党，依法行政、从严治政、廉洁从政，努力建设组织放心信任、人民满意信赖的政府。

　　（一）始终坚持正确的政治方向。坚决同以习近平同志为核心的党中央保持高度一致，牢固树立“四个意识”特别是核心意识、看齐意识，严守党的政治纪律和政治规矩，在思想上充分信赖核心、政治上坚决维护核心、组织上自觉服从核心、感情上深刻认同核心、行动上自觉看齐核心，更加扎实地把党的路线方针政策和各项决策部署落到实处。

　　（二）始终坚持全面从严治党。深入推进全市政府系统全面从严治党，落实主体责任，严肃党内政治生活，强化党内监督，维护山清水秀的政治生态。严格执行中央、省委和市委各项规定，严防“四风”反弹，用好数据铁笼，坚持无禁区、全覆盖、零容忍，从严监管公共资金、公共资源、公职人员，大力整治庸政、懒政、怠政，持续深化党风廉政建设和反腐败斗争。

　　（三）始终坚持以人民为中心的发展思想。树立群众观点、坚持群众路线，使政府作出的工作部署、出台的政策措施更加符合群众意愿，把更多的公共资源向民生领域倾斜。大力推行“互联网+政务服务”，让数据多跑腿、群众少跑路。认真解决涉及群众利益的热点难点问题，用心用情用力办实事、做好事、解难事，让群众更多更好地共享改革发展成果。

　　（四）始终坚持依法行政。把政府工作全面纳入法治轨道，切实做到法定职责必须为，法无授权不可为。抓好政务公开，推进决策公开、执行公开、管理公开、服务公开、结果公开。自觉接受人大法律监督、政协民主监督和社会监督、司法监督，广泛听取各民主党派、工商联、无党派人士意见。

　　（五）始终坚持实干担当。不断提升执行力，勇于做好证明题，市委有号召、政府有行动，市委有要求、政府有落实，健全抓执行落实的责任链和任务链，推动各项工作落细落小落实，以实实在在的工作成效证明市委决策部署的正确性。坚持问题导向、目标导向，认真研究问题、破解难题，迎难而上、排难而进，敢作敢为、善作善成。

　　各位代表，回顾过去，我们豪情满怀；展望未来，我们信心百倍。让我们更加紧密地团结在以习近平同志为核心的党中央周围，在省委、省政府和市委的坚强领导下，不忘初心、继续前进，坚守发展生态两条底线，建设更高水平全面小康，为加快打造创新型中心城市而努力奋斗，以优异成绩迎接党的十九大和省第十二次党代会胜利召开！
